# Bivalent-Like Chromatin Markers Are Predictive for Transcription Start Site Distribution in Human

**DOI:** 10.1371/journal.pone.0038112

**Published:** 2012-06-29

**Authors:** Zhihua Zhang, Xiaotu Ma, Michael Q. Zhang

**Affiliations:** 1 Department of Molecular Cell Biology, Center for Systems Biology, University of Texas at Dallas, Richardson, Texas, United States of America; 2 Center for Computational Biology, Beijing Institute of Genomics, Chinese Academy of Sciences, Beijing, People’s Republic of China; 3 Laboratory of Disease Genomics and Personalized Medicine, Beijing Institute of Genomics, Chinese Academy of Sciences, Beijing, People’s Republic of China; 4 Bioinformatics Division, Center for Synthetic and Systems Biology, TNLIST, Tsinghua University, Beijing, China; National Institutes of Health, United States of America

## Abstract

Deep sequencing of 5′ capped transcripts has revealed a variety of transcription initiation patterns, from narrow, focused promoters to wide, broad promoters. Attempts have already been made to model empirically classified patterns, but virtually no quantitative models for transcription initiation have been reported. Even though both genetic and epigenetic elements have been associated with such patterns, the organization of regulatory elements is largely unknown. Here, linear regression models were derived from a pool of regulatory elements, including genomic DNA features, nucleosome organization, and histone modifications, to predict the distribution of transcription start sites (TSS). Importantly, models including both active and repressive histone modification markers, *e.g.* H3K4me3 and H4K20me1, were consistently found to be much more predictive than models with only single-type histone modification markers, indicating the possibility of “bivalent-like” epigenetic control of transcription initiation. The nucleosome positions are proposed to be coded in the active component of such bivalent-like histone modification markers. Finally, we demonstrated that models trained on one cell type could successfully predict TSS distribution in other cell types, suggesting that these models may have a broader application range.

## Introduction

In eukaryotic organisms, gene transcription starts with the formation of a pre-initiation complex, followed by RNA polymerase II (Pol II) recruitment, initiation, promoter clearance, elongation and termination. Pol II often pauses after elongating a short distance (∼25–50 nt) until additional signals dictate that it escapes the pausing status and transfers to productive elongation (see [1] for a review). Transcription is initiated at most mammalian genes irrespective of their activity status, and transcriptional initiation is not just limited to active promoters [2], [3]. Transcriptional initiation is not a static process, as indicated by high-throughput Cap Analysis Gene Expression (CAGE) experiments which revealed that transcription does not always initiate from a single, fixed transcription start site (TSS). Instead, it could be started from a number of putative sites in the core promoter region of a gene. The probability of a site being chosen as an actual initiation location does not necessarily evenly distribute among all possible TSSs. Moreover, the distribution of TSSs in a core promoter is remarkably varied among genes [4]–[6], and this variation has been associated with the tissue-specificity of a gene’s expression in mammals [6]. Thus, the distribution of TSSs is tightly regulated. Indeed, accumulating evidence has indicated that the regulators of TSS distribution lie at both genetic and epigenetic levels [4]–[9], forming a multilevel regulatory network.

The internal structure of this multilevel regulation network, *i.e*. the relationship between histone modifications, nucleosome structure, Pol II status, and the TSS distribution, remains to be elucidated. Some *Drosophila* studies have suggested that static elements such as local DNA sequences, are major players in regulation of TSS distribution [8]; while other studies in human and fly have shown a strong association between TSS distributions and nucleosome organization in the proximal promoter regions [9], and even for the nucleosome organization *per se,* the causality of genetic and epigenetic cues is under debate. On the one hand, it has been clearly shown in yeast that nucleosome organization can be determined by static DNA sequences ([10], [11]. On the other hand, in fly and human nucleosome organization cannot be fully explained by DNA sequence alone, and increasing data have suggested that histone modifications, nucleosome remodelers, and Pol II status may also be important [12], [13]. Histone modification levels have been associated with and accordingly modeled to predict gene expression levels [14], [15]. For example, the tri-methylated form of histone H3 at lysine 4 (H3K4me3) is believed to be a marker of the active core promoters [16], while the tri-methylated form of histone H3 at lysine 36 (H3K36me3) is a marker of actively transcribed regions [16]. Thus, similar to gene expression level prediction, finding a model that quantitatively associates histone modifications, nucleosome organization and TSS distribution is of much interest. However, to accurately estimate the effect of histone modification on TSS distribution is not trivial, given the strong association between histone modifications and nucleosome positions [12].

Traditionally, promoter prediction has focused on the location of TSSs. As the TSS for a given promoter is not unique, the location must be described by a probability distribution function. An advanced promoter prediction model should not only predict the mode but also the shape of the distribution. As a first step towards this goal, this paper addresses whether the “width” of the distribution, *i.e.*, the variability of the TSS, can be predicted reliably. We utilize the Shannon entropy of the TSS distribution as a measure of the distribution “width”. We have derived several linear models for entropy prediction from different combinations of regulatory features, including histone modifications, transcription factor binding site scores and nucleosome accessibility levels. The analysis of our models reveal a clear pattern which suggests that those models which combine both active and repressive chromatin markers are much more predictive for the entropy of the TSS distribution than models with only single-type markers. This result suggests a new type of bivalent-like chromatin code associated with TSS distribution. Furthermore, histone modifications not only complement the information on nucleosome positions, but also encode additional information about the TSS distribution. Finally, we show that our model can successfully be used to predict the variability of initiation sites in other cell types, suggesting that we have extracted a general relationship between the regulatory elements and the TSS distribution.

## Results

The TSS distribution in mammals was empirically classified into four categories (single peak, broad, multi-modal, and broad with dominant peak [4]). This classification system was obtained from high-quality CAGE experimental data. Like all other next-generation sequencing technologies, CAGE data are subjected to sequencing noise [17]. Thus, instead of the standard deviation, we preferred use the Shannon entropy of the TSS distribution as the measure of the variability of TSS (“width”). Accordingly, we performed *in silico* simulations to show that entropy as measure of variability is a more robust measure against sampling noise than the standard deviation (See Text S1, Figure S1 and S2). By assuming that the TSSs in a core promoter region follow a Gaussian distribution, we treated the CAGE experiments as a sampling process in which reads are sampled from an unknown Gaussian population. An algorithm was developed to estimate the entropy of the Gaussian distribution from the sampled reads (see the Methods for the detailed algorithm). We limited our study to promoters with more than 10 reads because the theoretical universal convergence rates of entropy, 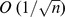 [18], will no longer substantially decrease when *n* is larger than 10.

### Nucleosome Position, Histone Modification Levels, and DNA Sequence Features are all Predictive of the TSS Distribution

**Figure 1:**
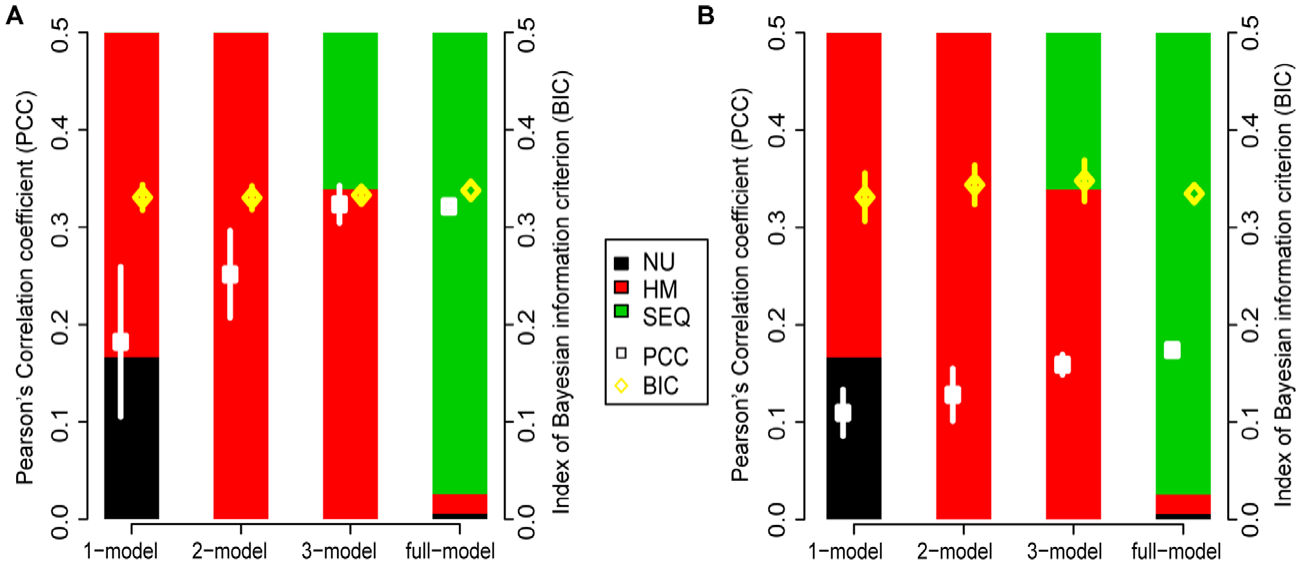
Features selected for the best models in HepG2. Stacked bars represent the distributions of selected feature types between nucleosome position (NU), histone modification levels (HM), and DNA sequence information (SEQ). The bar length represents the selected fraction of each type of features (with the range from 0 to 1). Squares and diamonds represent the mean PCC and the mean BIC of the best models in the corresponding model categories, respectively, A) for CpG-related promoters, and B) for nonCpG-related promoters.

In addition to CpG density [4]–[7], [19], nucleosome positions have been shown to be predictive of TSS distribution [9]. However, it is still unclear whether this is also true for histone modifications or transcription factors. To address this question, we collected publicly available data for transcript factor binding sites, nucleosome positions, and histone modifications in six human cell types (see Methods). In total, 180 features were pooled and examined in our analysis. We analysed CpG and non-CpG promoters separately, similar to previously applied approaches [15], [20]. This is because 1) we were interested in features other than CpG density, 2) previous studies have shown that CpG and non-CpG related promoters have distinctly different sequence features [21], and 3) distinguished histone modification profiles were found around the two types of promoters in human and mouse [22]. Similar to Karlic *et al*’s work which modeled gene expression from histone modification levels by linear regression models [14], we predicted the entropies of the TSS distributions by linear regression models and assessed the predictive power of the models by cross validation. Briefly, for a given set of features, these models take linear combinations of features as input and predict the TSS distribution entropy as the output. We performed the following steps to achieve a 5-fold cross validation. First, the dataset was separated into 5 partitions, taking four of the five partitions as the training set to learn parameters. Second, the obtained models were applied to the remaining data partition to predict TSS distribution entropy. This process was repeated 5 times for different combinations of 5 data partitions. Again, like Karlic *et al*. [14], we evaluated the performance of the model using Pearson’s Correlation Coefficient (PCC) between predicted and measured entropy. Finally, the average of the five PCCs was taken as the predictive power of a given combination of features. A high PCC indicates that the corresponding features have good predictive power and the five-fold cross-validation ascertains that the possible quantitative relationship revealed by the model is not limited to a subset of genes. The model derived with all the features (we refer to it as “full-model”) was significantly correlated to the TSS entropy (*r_full_*  = 0.41, p-value of t-test <1.6e-22; Figure 1 and Figure S5), demonstrating that the features in the pool are correlated with TSS entropies. The p-values of correlations in the rest of this paper are all <1.6e-22 (see Table S1), and have therefore been omitted from the text. The statistical significance of the best models was evaluated by comparing to two negative controls (Text S1and Figure S3).

We determined how many features were sufficient to predict entropies. Two lines of evidence showed that no more than 3 features appeared to be sufficient to build a linear regression model approaching the upper boundary of performance. First, the average performance of the best models (those with PCC >0.1) increased as the number of inputs increased, this performance increases between 1-models and 2-models is statistically significant (student’s test, P = 2.3e-8 and P = 0.025 for the models trained in CpG- and nonCpG-related promoters in HepG2 cells, respectively). However, when the number of inputs increased beyond 3, the PCC did not demonstrate any further increase, since models with 3 inputs had already reached about 95% of the performance level of the full-model in all cell types that we examined (Figure 1). Second, we used the Bayesian information criterion (BIC) to test if increasing the model complexity (the number of inputs in the linear regression model) would be beneficial [23]. The BIC is a criterion for model selection among a finite set of models by introducing a penalty for the number of parameters in the model. If increasing complexity benefits modeling, BIC will decrease. However, with the exception of the full-model in human stem cells, we observed no significant reduction in BIC with increasing number of features (Figure 1). Thus, as few as three inputs features at the promoter were enough to faithfully model TSS entropies, and in the remaining part of the paper we will focus on models involving 1, 2 and 3 input features (referred to as 1-, 2-, and 3-models in this paper, respectively; Table S1).

**Figure 2:**
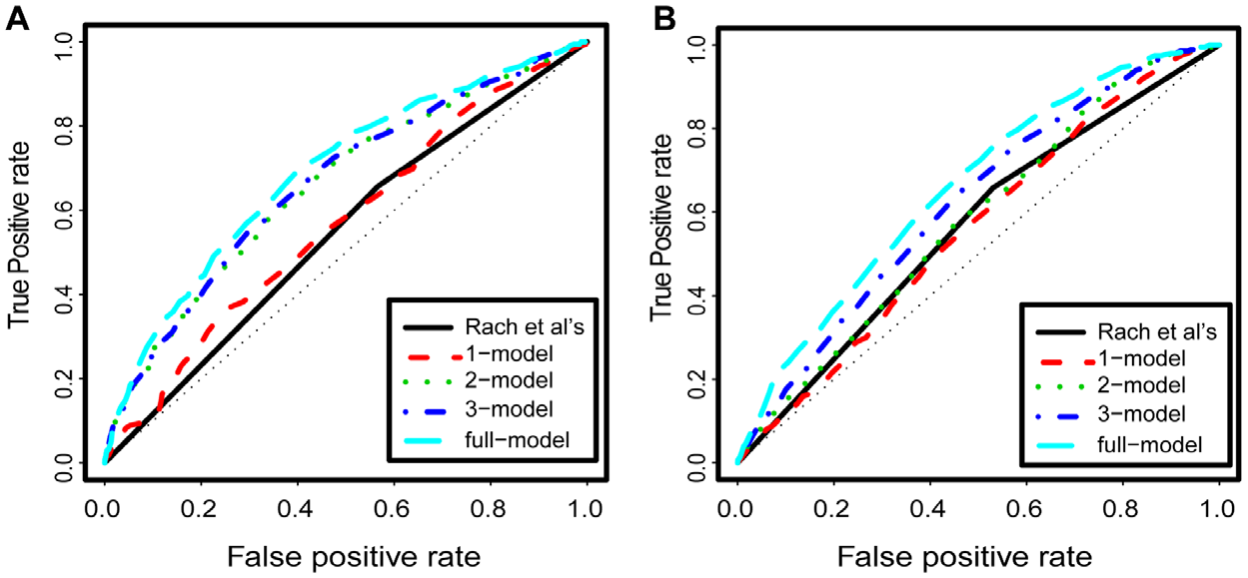
The receiver operating characteristic (ROC) curve for the performance of models trained in NHEK cell, and the performance of *Rach et al*’s logistic regression model. A) for CpG-related promoters, and B) for nonCpG-related promoters.

For the best 1-models, the selected features are expected to be the most predictive. In all six cell types, these selected features were mostly comprised of nucleosome positions or a few histone modification levels as the best predictors (Figure 1). The histone modification types selected by our best 1-models were those features that were highly correlated with the nucleosome position. As a comparison, our best 1-models performed similarly to the *l1-*logistic model developed by Rach and colleagues [9] in classifying the TSS distribution of promoters as “narrow peak” and “broad peak” (Figure 2 and Methods). Their *l1-*logistic model suggested the importance of nucleosome positions for the TSS distribution [9], and an analysis of our best 1-models further supported this suggestion.

Notably, the best 2-models had significantly higher PCCs than the best 1-models (except for cell line GM12878), and the PCCs of our best 2-models could reach up to 80% of the upper boundary of performance level given by the full-model (Figure 1). The ROC curves were also better in the best 2-models as compared to our best 1-models and the *l1-*logistic model [9] (Figure 2). In the best 2-models, more than 90% of all the features selected were histone modification levels for all six cell types we examined (Figure 1). Remarkably, nearly half of the histone modification types selected by the best 2-models are highly correlated with nucleosome positions. Therefore, the information that is encoded in nucleosome structure for TSS distribution may be reflected by those histone modifications, as analyzed below. Moreover, additional predictive information may also be encoded in histone modifications for TSS distribution as the remaining histone modification types are poorly correlated with nucleosome positions.

For the best 3-models, less than 10% of improvement on the model performance levels was achieved compared to the best 2-models (Figure 2 and 1). Surprisingly, in all the six cell types examined, less than 30% of the selected features were DNA sequence motifs (Figure 1). In yeast, nucleosomes are intrinsically organized by static DNA sequences [10], [24]; however, in human it is less clear whether DNA sequence or epigenetic elements exert the stronger influence on nucleosome positioning [7], [25], [26]. Given the tight link between nucleosome position and TSS distribution shown above and by others [9], this result suggest that the transcription factor binding motifs included in the present models may not be direct regulators of TSS distribution in human.

### A Bivalent-like Chromatin Code at the Core Promoter Predicts TSS Distribution

We next asked what underlying information for TSS distribution prediction is encoded by histone modifications in the core promoter regions. One type of such information may be associated with nucleosome position (Figure 1). It has been shown that the nucleosome positions can be inferred from histone modification data [12]. To further study the link between the predictive histone modifications selected by our best models and nucleosome positions, we analyzed their relationship in the core promoter regions.

**Figure 3:**
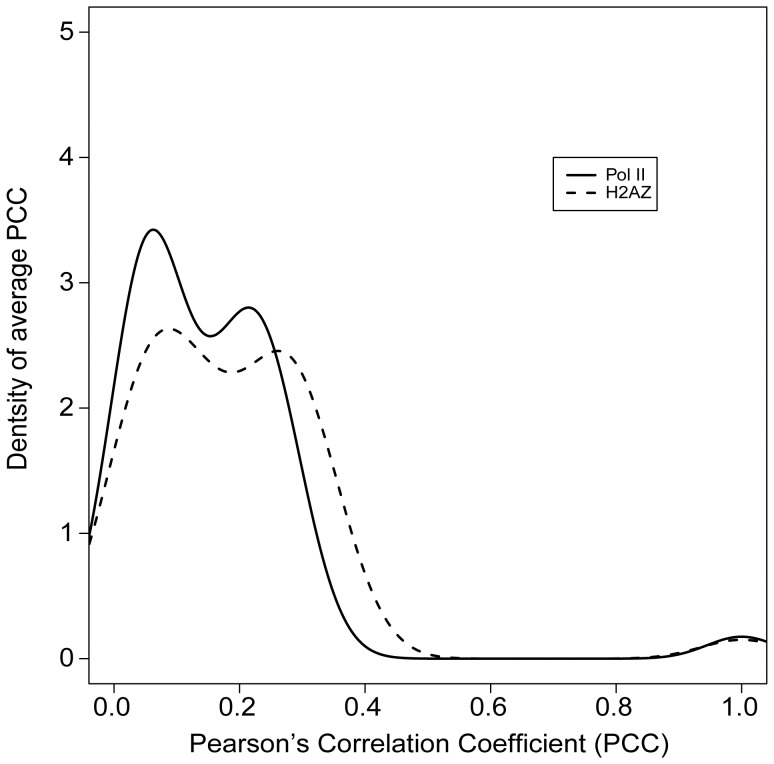
The distribution of PCCs among the 41 histone modifications, Pol II and H2A.Z levels in the promoter regions of CD4+ T cells.

In CD4+ T cells, we found that histone modifications could be classified into two categories according to their correlation with nucleosome positions in core promoter regions. We calculated the PCCs between histone modifications and nucleosome positions for the 41 histone modification types that have been mapped genome-wide in human CD4+ T cells [16], [27]. Promoter nucleosome positions for the same cell type were inferred from data on polymerase II (Pol II) binding or H2A.Z association levels. Both datasets were generated from Dr. Zhao’s lab [16], [28]. Pol II and H2A.Z were chosen as the reference markers for nucleosome positions because a strong correlation between these two markers and the bulk nucleosomes in the core promoter regions has been shown [28], [29]. The PCCs showed a clearly bimodal distribution (Figure 3), suggesting that the histone modifications could be classified into two categories. These two categories were then revealed by k-means clustering, and are subsequently referred to as Class I and II (Table S2).

**Figure 4:**
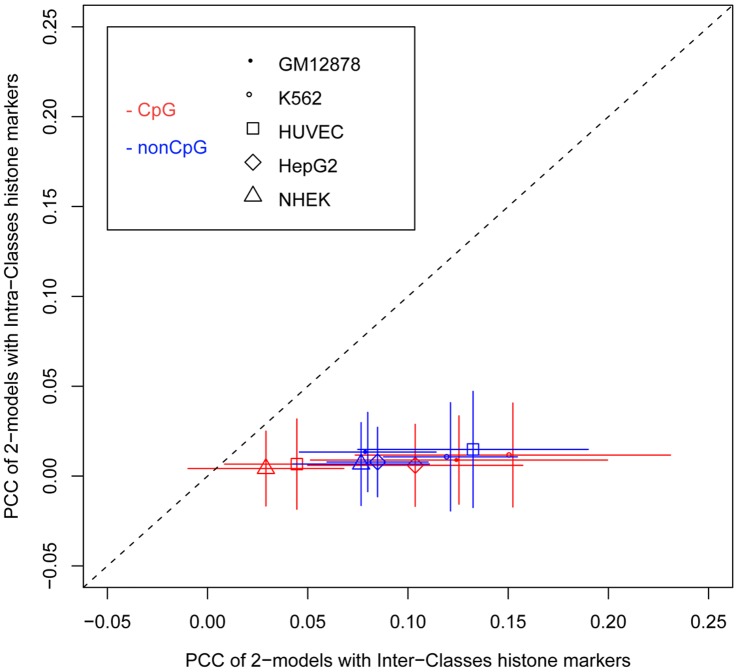
Predictive power of 2-models. The dots represent the predictive power of the models, blue and red indicating models were trained and tested in CpG-related and nonCpG-related promoters, respectively. The x-axis is the PCC generated by the 2-models involved in two histone modifications, one is from Class I, and the other is from Class II. The y-axis is the PCC generated by the 2-models involving two histone modifications from the same class, either Class I or Class II. Error bars give the standard derivations within the two cases.

We next assessed what possible underlying information for the TSS distribution prediction could be retrieved from such classification. The Class I histone modifications are enriched with “active” transcriptional markers frequently found in promoter regions (i.e, H2BK120ac, H3K27ac and H3K4me3), which are relatively correlated to nucleosome positions in the promoter region. On the other hand, Class II histone modifications are enriched with transcriptionally repressive markers (e.g. H3K27me3, H3K4me1, H3K79me3 and H4K20me1) [30], which are far less well correlated to nuclesome positions in the promoter region (Table S2). If nucleosome position is a major information associated with TSS distribution, we would expect that most of the histone modification types selected by the best 1-models to be from Class I. Indeed, 38 of 39 (97.4%) histone modifications selected by best 1-models belongs to Class I. Meanwhile, if nucleosome position is the *only* predictive information for TSS distribution, we would expect that most of the histone modification types selected by the best 2-models also to be from Class I. However, given that histone modifications are evenly distributed between class I and class II, it was surprising that only 76% (575 out of 757) of the histone modifications selected by the best 2-models were from Class I. This results would suggest most of the best models utilize the information content of both active and repressive histone markers. This trend was even clearer when we raised the threshold for the definition of best 2-models. Indeed, the predictive power of best 2-models that include both active and repressive histone markers was much higher than the best 2-models include only single-type histone markers (Figure 4). This result suggested that there is a bivalent-like chromatin code is associated with TSS distribution prediction in core promoter region. In the bivalent-like chromatin code, the active histone markers may be associated with the positions of nucleosomes in core promoter regions. We noticed that H3K36me3 and H4K20me1 in Class II signify transcriptional activity when present in the gene body regions [27]. To investigate if this could bias our analysis, we compared two group of models, the first group containing models that include both active and repressive histone markers from Class I and Class II, respectively, and the second group containing models that include Class I active histone markers and the Class II H3K36me3 or H4K20me1. We found that the performance of models in the first group is better than the performance of models in the second group (Figure S6). This result suggests that H3K36me3 or H4K20me1 did not introduce bias into the analysis we present above.

### The Associations between the Histone Modification Levels and TSS Distribution are General

We have shown that for a given cell type, models involving as few as 3 different types of histone modifications can approximate the predictive power of the full-model. Therefore, it was interesting to ask whether models trained on the data of one cell type could be used to predict the TSS distribution in another cell type. To assess this possibility, we applied the best 1-, 2- and 3-models trained in one cell type to all the other cell types in our dataset. As shown in Figure S4, the average predictive power of a given model across the cell types was similar to that of the cell type in which the model was trained. This result strongly suggested a general relationship between histone modifications and TSS distribution that is largely independent of the cell context.

In summary, we have found that histone modifications are associated with TSS entropy, which is a novel measure for TSS distribution. Our analysis revealed that only 3 features are sufficient to achieve reliable TSS entropy prediction. Our data suggested the existence of a bivalent-like chromatin structure for TSS distribution prediction in the promoter region, in which the information for nucleosome positions may be encoded. Moreover, the relationship could be generalized across different cell types indicating that the model is largely independent of the cellular context.

## Discussion

Associations between TSS distribution and DNA sequence elements [4]–[8], [19], and between these and nucleosome structure have previously been reported [9]. In this work, we aimed to quantitatively model TSS distribution using transcription factor binding sites, nucleosome organization and histone modification levels as inputs. We found that a very small number of features were responsible for most of the predictive power attainable by the models. A special subset of 2-models, that is, models including both active and repressive histone modification markers had considerably better predictive power than other models(Figure 4, S6), leading us to propose that there exists a bivalent-like chromatin control with substantial predictive power for TSS distribution.

Bivalent histone markers were first observed as the occurrence of high levels of both active H3K4me3 and repressive H3K27me3 in developmental genes in embryonic stem cells [31]. More recently, they have been found in hypermethylated genes in cancer [32], as well as in aging-associated DNA hypermethylated promoters in somatic cells [33]. In mice, it was reported that a bivalent chromatin pattern, in combination with neuronal factors, controlled the expression of a brain-specific gene *Grb10* [34]. Although the two histone markers H3K4me3 and H3K27me3 have, indeed, been identified as one pair of the best predictors in our models, the term bivalent-like we borrowed here does not specifically refer to this particular histone marker pair. Rather, we have emphasized, in a general sense, that the combinatorial pattern of both active and repressive histone markers is associated with TSS distribution prediction. Based on the limited amounts of data on histone modification types in the cell types studied, we cannot eliminate the possibility that other histone markers may also be predictive of TSS distribution, and the observed bivalent-like pattern need therefore to be corroborated by further data. Interestingly, the finding of bivalent-like pattern presented here is consistent with the recent finding that most *cis*-regulatory modules include both acting and repressive regulators [35].

There are other possible information types which might be also encoded in the bivalent-like histone modification patterns for TSS distribution. One such type information might be linked to stalled Pol II. Several lines of evidence support the linkage between the stalled Pol II and TSS distribution. For example, *GAGA* and the pause button motifs were found to be enriched in peaked promoters [8]. In *Drosophila*, stalled Pol II has been observed with well-positioned TSSs [36]. Pausing Pol II could maintain accessibility to the promoter region [37], or it could prevent the formation of repressive chromatin [38]. It has also been proposed that the pausing Pol II could serve as a checkpoint for coupling transcription and mRNA processing, the pre-mRNA thus waiting for the desired modification patterns to be formed in the downstream exonic regions [39]. In addition to pausing Pol IIs, we also noticed that one modification, H4K20me1, was selected in most of the best 2-models. H4K20me1 can act as repressive modification [40], [41], however, it has also been observed in the promoter and gene body region of actively transcribed genes [16], [27]. Moreover, it is one of the most predictive histone modification types for gene expression level [14]. Given the complex role of H4K20me1 with regard to transcription, we may hypothesize the existence of multiple code readers for this same histone modification type under different situations. For example, H4K20me1 interacts with Lethal 3 malignant brain tumor 1 (*L3MBTL1*) [42] and *JMJD2A* [43]. Because of the potential variation in binding of factors under different conditions or cell types, it is reasonable to speculate that H4k20me1 could affect transcription by affecting the structure and properties of nucleosomes, or, alternatively, by influencing the properties of protein binding on the nucleosomes which, in turn, would affect transcription. The two possibilities are not necessarily mutually exclusive, *i.e*. the exposure of a cryptic binding site may be a consequence of a change of nucleosome conformation. Therefore, it is easy to imagine that this may results in the activation of transcription in one case and the repression of transcription in another [44]. Given 1) the complicated network of interactions among transcription factors, transcription initiation, nucleosome positions, and DNA replication [45], and 2) the fact that even the best full-model only capture less than 50% of the variation of TSS entropy, it is clear that more sophisticated data and modeling is needed to improve our understanding of TSS distribution and the regulation of transcription initiation.

## Materials and Methods

### Data

Histone modification data for CD4+ T cells were retrieved from the published mapping [16], [27], and the nucleosome position data of the cell types were retrieved from Dr.Zhao’s lab [28]. Data for the GM12878, K562, hESC, HUVEC, HepG2, and NHEK cell lines were retrieved from the ENCODE Project [46], in which histone modifications were mapped by the Broad Institute [31], DNase I hypersensitivity data were produced by the University of Washington [47], and CAGE data were generated by the RIKEN institute [48].

We downloaded the human reference genome (Hg18) and retrieved RefSeq gene annotations from the UCSC Genome Browser (http://genome.ucsc.edu). Transcription factor binding sites (TFBSs) were scanned in all analyzed core promoter regions by the STORM software [49], using the known position weight matrices annotated by TRANSFAC [50]. The threshold for TFBS identification by STORM was a P-value <1e-5. The core promoter regions were defined as 1000 base pairs (bp) upstream to 1000 bp downstream of the annotated TSS in the RefSeq genes. The transcription factor binding motifs were further clustered into 165 clusters by a Bayesian motif clustering algorithm to reduce the motif redundancy [51], and the TF binding affinities for the motifs in a single cluster were combined in the subsequence analysis. The clusters of transcription factor binding motifs can be found in Table S3.

### Estimation of the Entropy of the TSS Distribution

By assuming that the TSSs in a promoter follow a Gaussian distribution with an unknown standard deviation, we defined the entropy of the TSS distribution of this promoter as the entropy of this Gaussian distribution:
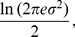
(1)where *σ* is the unknown standard deviation of this Gaussian distribution. We estimate tag entropies from the CAGE data. One way to estimate the entropy is to estimate the standard deviation of this Gaussian distribution by taking the standard deviation of observed CAGE tags. However, as we show in the Text S1, the standard deviation estimation is not a robust against sequencing noise or depth. Therefore, an alternative is to directly take the observed CAGE tag distribution curve as an approximation of the probability density function curve, known as a histogram estimator:
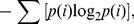
(2)where p(i) denotes that the frequency of tags has been observed in bin i. A similar estimation was recently used in a study on Drosophila [8]. However, it is well known that this histogram estimator is biased [52], and although attempts to correct such bias have been made, they may not always be satisfactory for general use [52]. To overcome this systematic bias [52], an algorithm has been developed to adjust this estimated tag entropy as follows:

Step one, we built a reference matrix, termed the SDEM. In the SDEM, each entry represents a particular Gaussian and sampling scenario. For example, row *i* and column *j* corresponded to the scenario in which *i* tags have been sampled from a Gaussian distribution with *σ  =  j.* The content in the entry is the mean and standard deviation of the estimated entropies by formula (2), for a scenario in which the calculation is based on resampling 50 times. The CAGE experiments were simulated as draw samples from a given Gaussian population. The sequencing depths were simulated as the number of samples drawn from the population. Because we were not interested in promoters with a flat TSS distribution, we only simulated Gaussian populations with *σ* in the range [0,100]. When the sample size is larger than 50, the estimation has been found to be sufficient. In our simulations, the number of samples ranged from 1 to 200. Therefore, the SDEM is a 200×100 matrix.

Step two, for any given promoter with *k* real CAGE tags sequenced, we first calculated an unadjusted entropy *U* by formula (2). Then, *U* was used to calculate the likelihoods of entropy distributions for all the entries in the *k-th* row of the SDEM. We chose the scenario which has the maximal likelihood for *U* as our predicted Gaussian distribution of TSSs in this promoter. For any given SDEM scenario, the likelihood of an entropy distribution is the integral of the probability distribution function in the neighborhood of *U*, [*U* − α, *U* + α], where α  = 0.1. We tested other values of alpha without significant changes to our results. The adjusted entropy was then calculated by formula (1) using the predicted Gaussian distribution. We denoted this adjusted entropy as the TSS entropy for this promoter. The matrix SDEM of mean and standard deviation can be found online as the Text S2 and S3, respectively.

### Linear Regression and l1-logistic Classification

We used the *lm()* function in the R package (www.r-project.org) to perform linear regression. The sum of ChIP-seq reads in the promoter region were used as features representing the level of histone modifications and DNase hypersensitivity, and the sum of STORM scores for each cluster were used to represent the TFBS cluster feature. The P-values, BIC, intercepts, and coefficients for the best 1-, 2- and 3-models can be found in Table S1. The *l1-*logistic classification was performed by the *l1*_logreg package [53].

## Supporting Information

Figure S1 **Comparison between TSS entropy’s and STD’s ability to distinguish two Gaussian populations with a uniform noise background.**
(TIF)

Figure S2 **Comparison between TSS entropy’s and STD’s ability to distinguish two Gaussian populations with a Gaussian noise background.**
(TIF)

Figure S3 **Performance distribution of best 2-models for K562 cells.**
(TIF)

Figure S4 **The receiver operating characteristic (ROC) curve for the performance of models trained in GM12878 and applied in NHEK cells.**
(TIF)

Figure S5 **Features selected for the best models.**
(TIF)

Figure S6 **Predictive power of 2-models.**
(TIF)

Table S1 **The models of 1-, 2-, 3- and full-models.**
(XLSX)

Table S2 **Two classes of histone modifications.**
(DOCX)

Table S3 **Clusters of Transcription factors.**
(XLSX)

Text S1 **Supporting discussions on sequencing noise and negative control.**
(DOCX)

Text S2 **The reference matrix SDEM of means.**
(TXT)

Text S3 **The reference matrix SDEM of standard deviations.**
(TXT)
