# Supplementary figures and images for: Bivalent-Like Chromatin Markers Are Predictive for Transcription Start Site Distribution in Human
Source: PLoS One. 2012 Jun 29;7(6):e38112. doi: 10.1371/journal.pone.0038112 (PMC3387189; doi:10.1371/journal.pone.0038112)

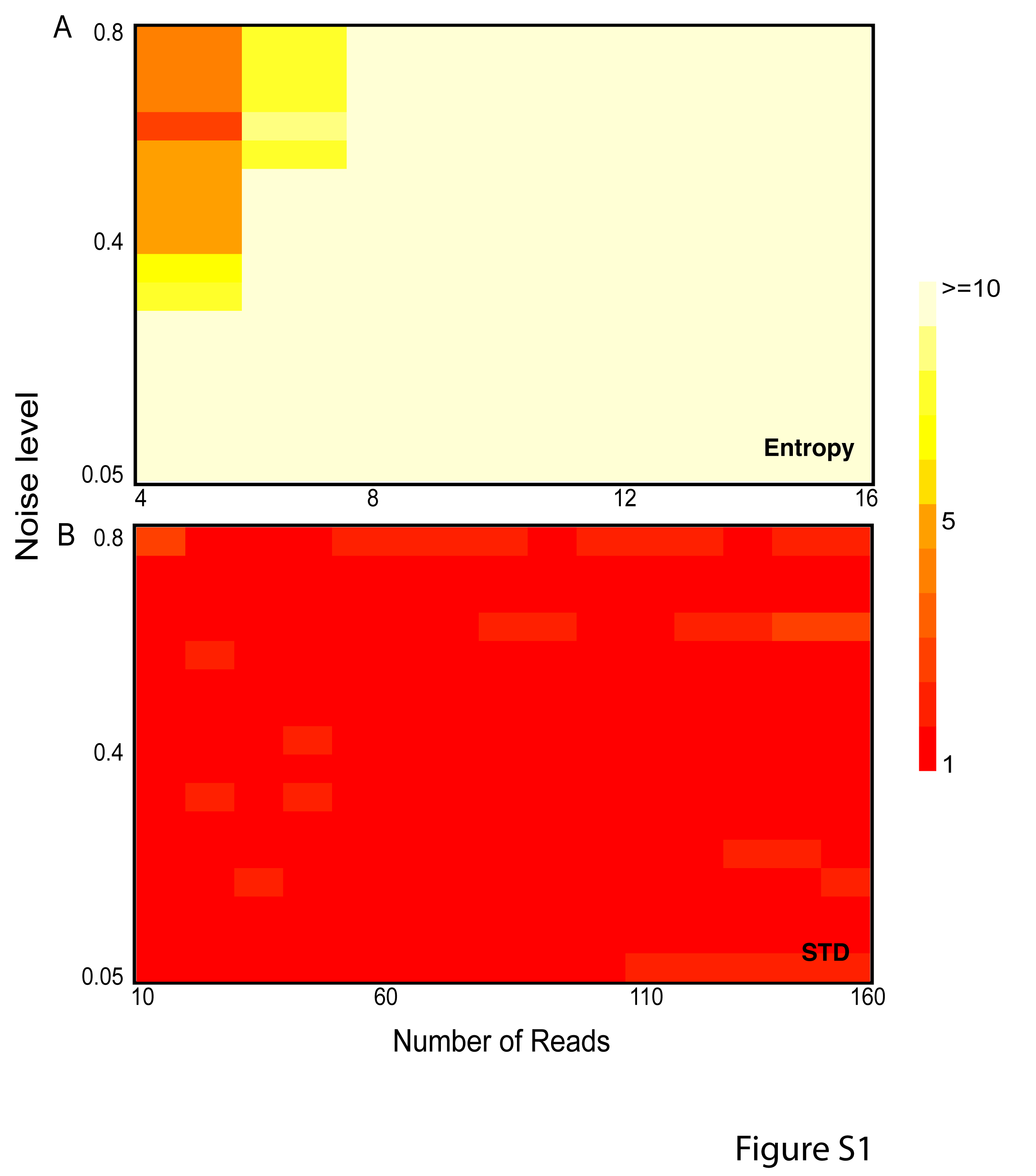

Supplement: Figure S1 — Comparison between TSS entropy’s and STD’s ability to distinguish two Gaussian populations with a uniform noise background. (TIF) [file pone.0038112.s001.tif]

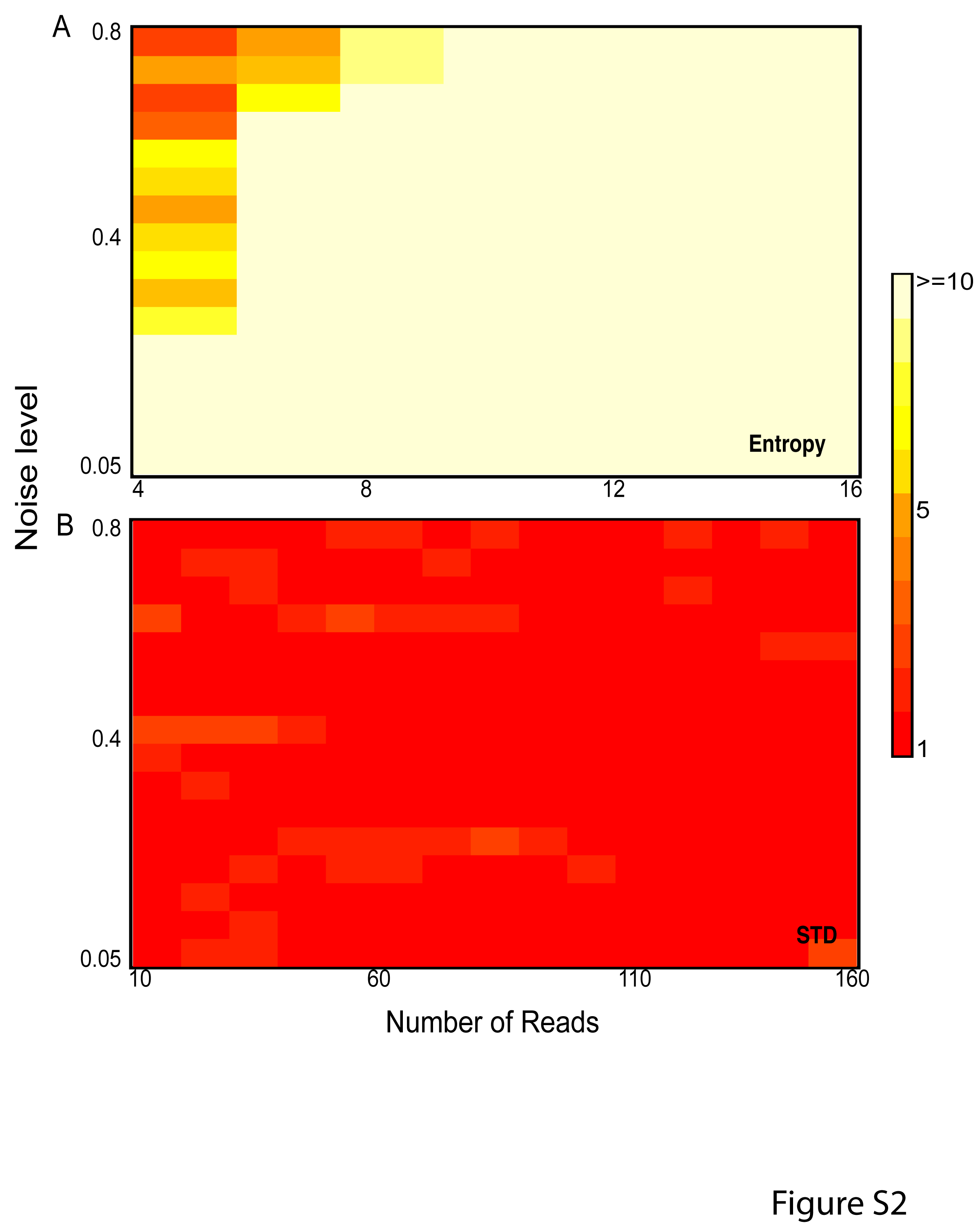

Supplement: Figure S2 — Comparison between TSS entropy’s and STD’s ability to distinguish two Gaussian populations with a Gaussian noise background. (TIF) [file pone.0038112.s002.tif]

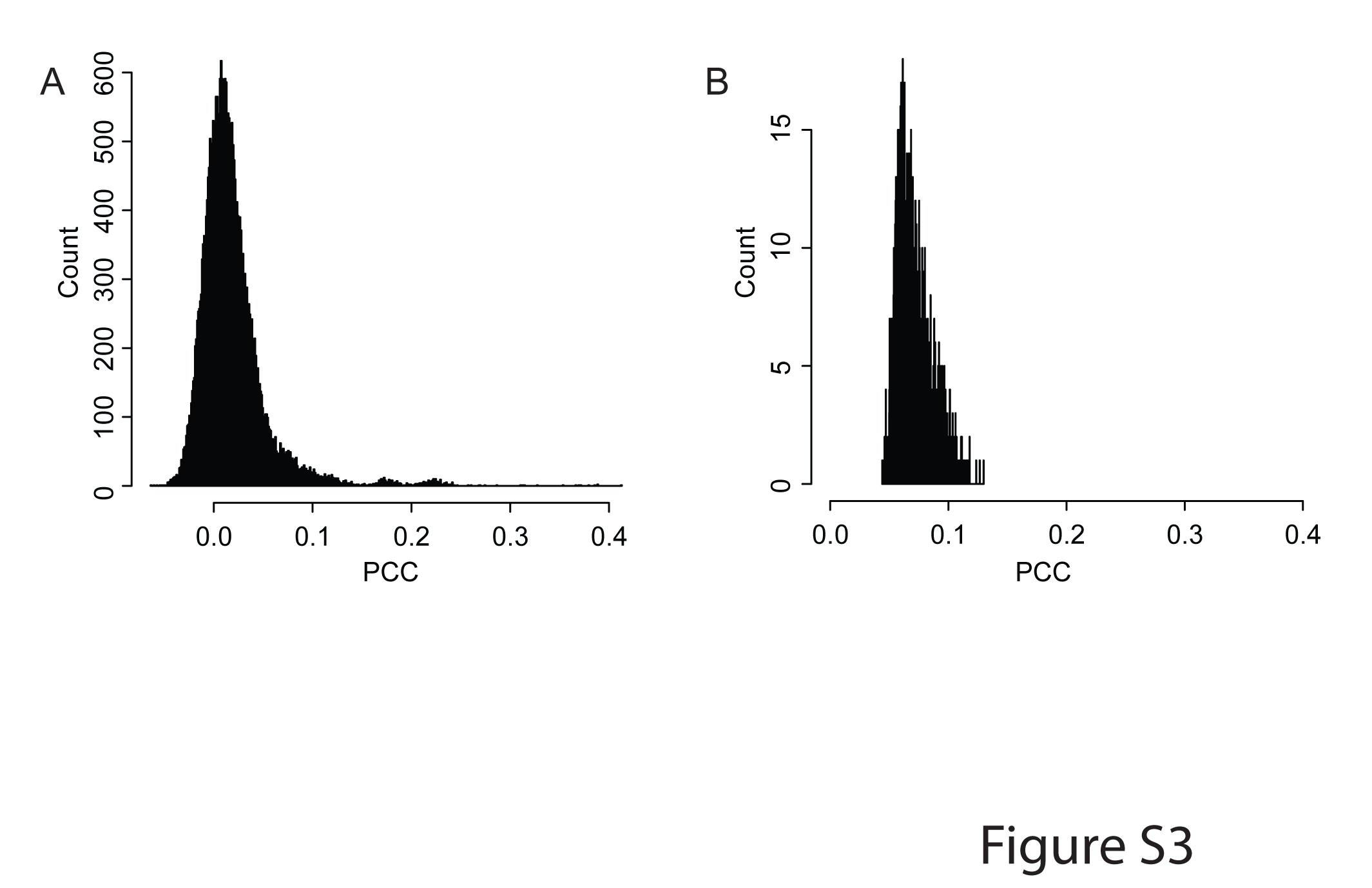

Supplement: Figure S3 — Performance distribution of best 2-models for K562 cells. (TIF) [file pone.0038112.s003.tif]

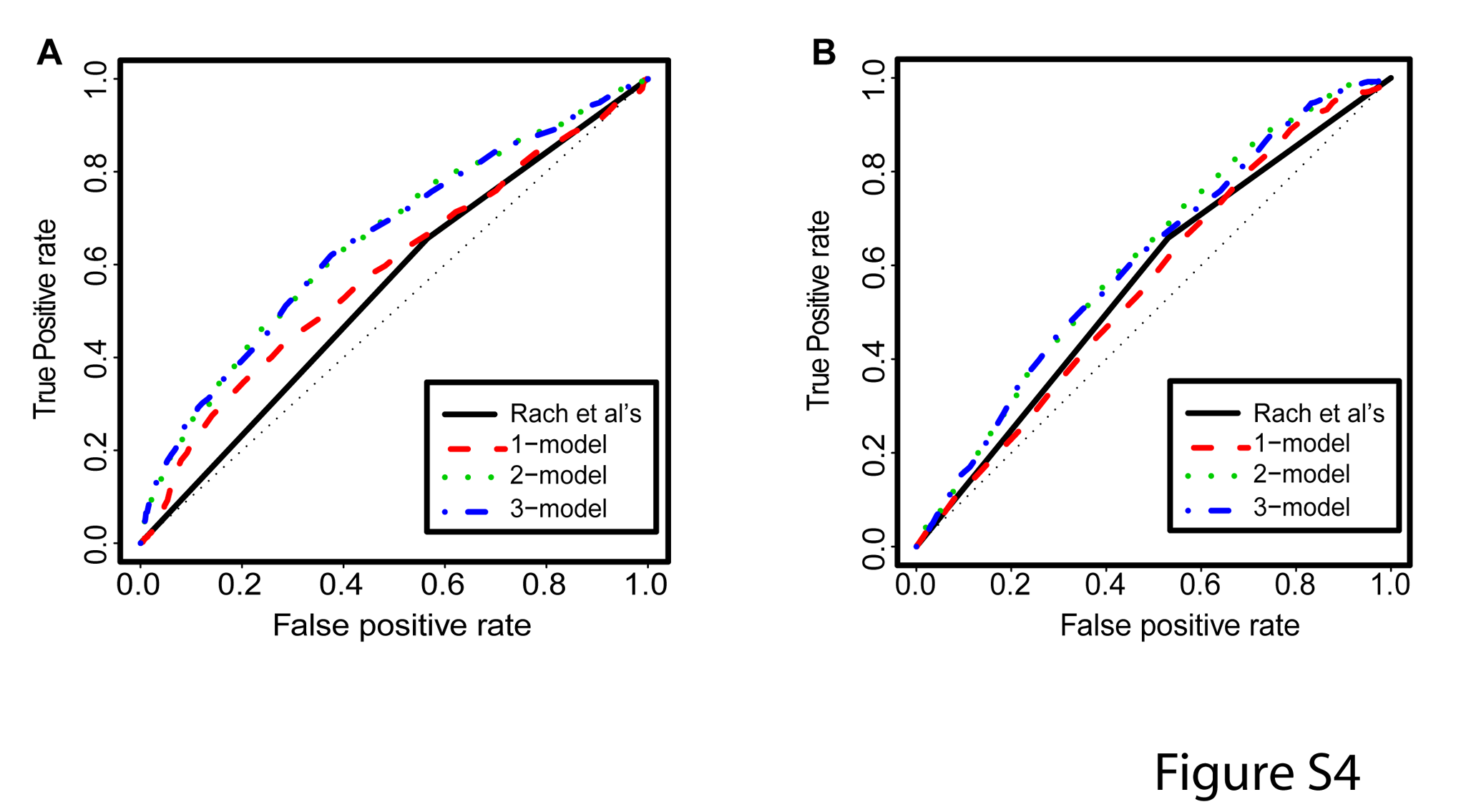

Supplement: Figure S4 — The receiver operating characteristic (ROC) curve for the performance of models trained in GM12878 and applied in NHEK cells. (TIF) [file pone.0038112.s004.tif]

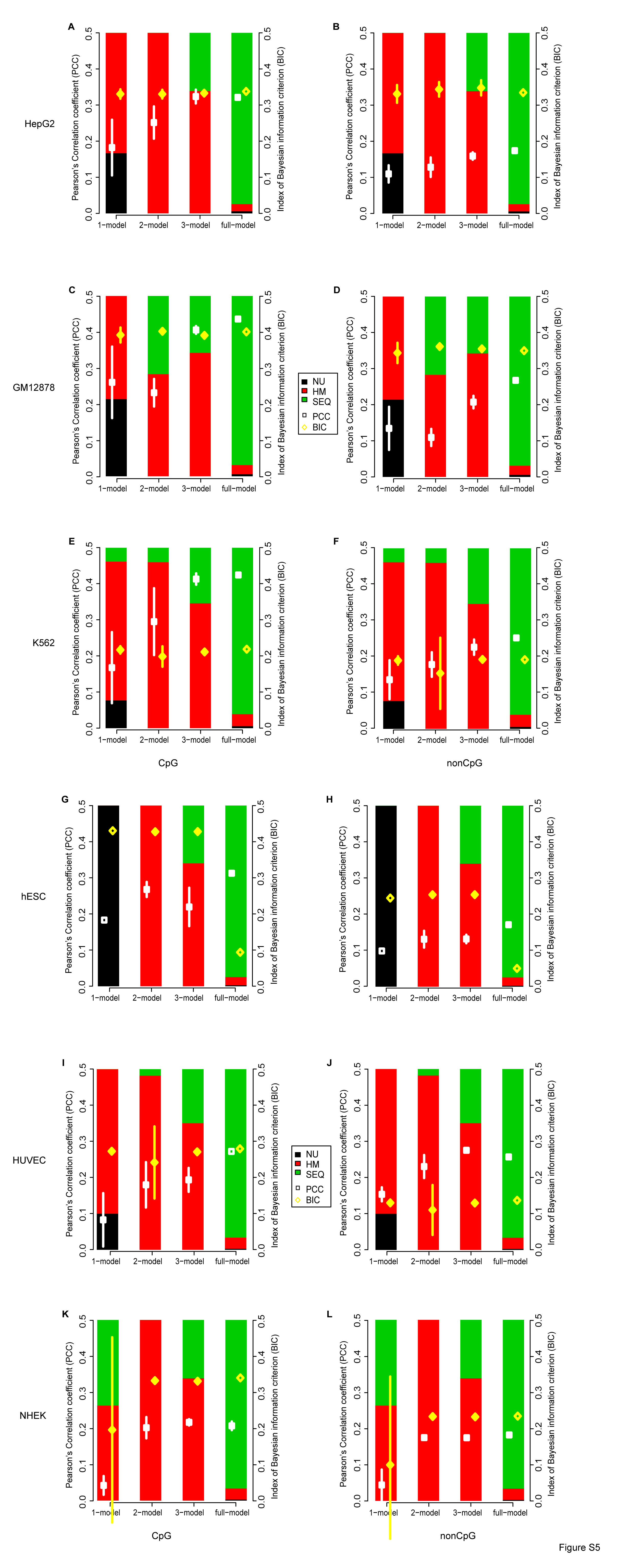

Supplement: Figure S5 — Features selected for the best models. (TIF) [file pone.0038112.s005.tif]

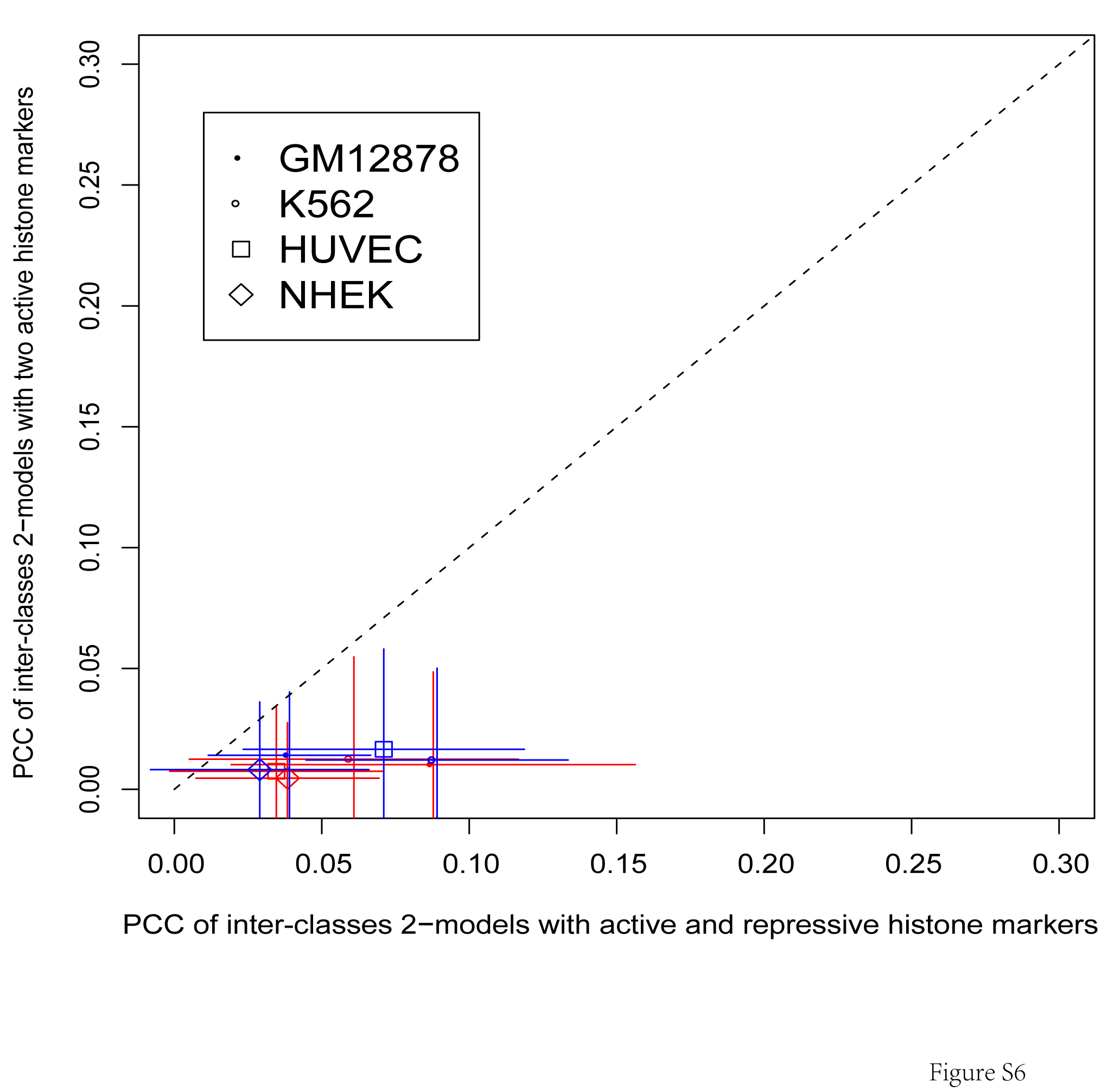

Supplement: Figure S6 — Predictive power of 2-models. (TIF) [file pone.0038112.s006.tif]
