# Supplementary material for: Bivalent-Like Chromatin Markers Are Predictive for Transcription Start Site Distribution in Human
Source: PLoS One. 2012 Jun 29;7(6):e38112. doi: 10.1371/journal.pone.0038112 (PMC3387189; doi:10.1371/journal.pone.0038112)
Supplement: Table S2 — Two classes of histone modifications. (DOCX) [file pone.0038112.s008.docx]

| HM type | by H2AZ | by Pol II | PCC to H2AZ | PCC to Pol II |
| --- | --- | --- | --- | --- |
| H2BK120ac | 1 | 1 | 0.288 | 0.234 |
| H2BK12ac | 1 | 1 | 0.215 | 0.180 |
| H2BK20ac | 1 | 1 | 0.267 | 0.222 |
| H2BK5ac | 1 | 1 | 0.301 | 0.253 |
| H3K18ac | 1 | 1 | 0.349 | 0.265 |
| H3K27ac | 1 | 1 | 0.326 | 0.271 |
| H3K36ac | 1 | 1 | 0.243 | 0.206 |
| H3K4ac | 1 | 1 | 0.265 | 0.228 |
| H3K9ac | 1 | 1 | 0.270 | 0.221 |
| H4K16ac | 1 | 1 | 0.199 | 0.188 |
| H4K5ac | 1 | 1 | 0.273 | 0.189 |
| H4K8ac | 1 | 1 | 0.298 | 0.194 |
| H4K91ac | 1 | 1 | 0.312 | 0.265 |
| CTCF | 1 | 1 | 0.214 | 0.248 |
| H3K4me1 | 1 | 2 | 0.145 | 0.077 |
| H3K4me2 | 1 | 1 | 0.266 | 0.176 |
| H3K79me3 | 1 | 2 | 0.023 | 0.065 |
| H4R3me2 | 1 | 2 | 0.040 | 0.014 |
| H2AK5ac | 2 | 2 | 0.022 | 0.000 |
| H2AK9ac | 2 | 2 | 0.162 | 0.138 |
| H3K14ac | 2 | 2 | 0.114 | 0.067 |
| H3K23ac | 2 | 2 | 0.144 | 0.067 |
| H4K12ac | 2 | 2 | 0.166 | 0.141 |
| H2AZ | 2 | 2 | 1.000 | 0.241 |
| H2BK5me1 | 2 | 2 | 0.056 | 0.074 |
| H3K27me1 | 2 | 2 | 0.104 | 0.045 |
| H3K27me2 | 2 | 2 | 0.065 | 0.034 |
| H3K27me3 | 2 | 2 | 0.093 | 0.056 |
| H3K36me1 | 2 | 2 | 0.133 | 0.098 |
| H3K36me3 | 2 | 2 | 0.000 | 0.020 |
| H3K4me3 | 2 | 1 | 0.351 | 0.301 |
| H3K79me1 | 2 | 2 | 0.041 | 0.056 |
| H3K79me2 | 2 | 2 | 0.007 | 0.039 |
| H3K9me1 | 2 | 1 | 0.251 | 0.177 |
| H3K9me2 | 2 | 2 | 0.070 | 0.056 |
| H3K9me3 | 2 | 2 | 0.046 | 0.054 |
| H3R2me1 | 2 | 2 | 0.082 | 0.071 |
| H3R2me2 | 2 | 2 | 0.058 | 0.049 |
| H4K20me1 | 2 | 2 | 0.015 | 0.073 |
| H4K20me3 | 2 | 2 | 0.088 | 0.125 |
| PolII | 2 | 2 | 0.241 | 1.000 |
