# Supplementary material for: Bivalent-Like Chromatin Markers Are Predictive for Transcription Start Site Distribution in Human
Source: PLoS One. 2012 Jun 29;7(6):e38112. doi: 10.1371/journal.pone.0038112 (PMC3387189; doi:10.1371/journal.pone.0038112)
Supplement: Text S1 — Supporting discussions on sequencing noise and negative control. (DOCX) [file pone.0038112.s010.docx]

Supplemental Text for

**Bivalent-like chromatin markers are predictive for transcription start site distribution in human**

Zhihua Zhang1,2,3, Xiaotu Ma1, Michael Q Zhang 1,§

1. Department of Molecular Cell Biology, Center for Systems Biology, University of Texas at Dallas, 800 W Campbell Road, Richardson, TX 75080.
2. Center for Computational Biology, and
3. Laboratory of Disease Genomics and Personalized Medicine, Beijing Institute of Genomics, Chinese Academy of Sciences, Beijing 101300, China;

§ Corresponding author.

Tel: +10-972-883-2523

Fax: +10-972-883-5710

Email: [michael.zhang@utdallas.edu](mailto:mzhang@cshl.edu)

**A sequencing noise resistance measure for TSS distribution**

Previously, TSS distribution was empirically classified into four categories by the shape of CAGE tag clusters (single peak, broad, multimodal, and broad with dominant peak; see (Carninci et al. 2005; Carninci et al. 2006; Kawaji et al. 2006)). Although the CAGE experiments in the Fantom project were not sequenced as deeply as in other high-throughput sequencing technology (Xu et al. 2010), it still subject to sampling effects. Moreover, because the sequencing depth of the ENCODE projects CAGE experiments was deeper than that in Fantom project it is important to control for such effects of sequencing noise in CAGE. Therefore, in this work, we introduced a novel sequencing noise resistance measure for TSS distribution, termed TSS entropy. TSS entropy is defined as follows. Assuming that the real TSS follows a Gaussian distribution, we treated CAGE as a process of sampling from an unknown Gaussian population. An algorithm was developed to estimate the entropies of the Gaussian distribution from the sampled reads (see Methods).

To show that TSS entropy is a measure of resistance to sequencing noise, we compared it with the standard deviation (STD). There are at least two types of noise known to affect CAGE experiments: one is caused by general experimental fluctuation, and the other is caused by recapped cleaving RNAs (Schoenberg and Maquat 2009) that can lead to undesired CAGE peaks near promoter regions (Hoskins et al. 2011). We modeled the general experimental fluctuation and recapping RNAs by an additional uniform noise background and Gaussian noise sources, respectively. In both noise conditions, two Gaussian populations can be easily distinguished by comparing TSS entropies, even at noise-to-signal ratio levels as high as 80%, as long as sufficient read coverage is achieved (Figure S1 and S2). On the other hand, since STD is extremely sensitive to either type of noise, as little as 0.5% of the noise could diminish the ability to distinguish two Gaussian populations using STD (Figure S1 and S2), and such diminishment could not be rescued by simply increasing read coverage within the range we examined. Therefore, when the sequencing depth, or read coverage, was high enough, the estimate of TSS entropy was highly resistant to sequencing noise. Based on the simulations described above, we limited our study to promoters which had at least 10 CAGE tags.

**Negative control**

To evaluate if the best models we chose were truly predictive, the following two facts served as negative controls. First, most models were not predictive. In other words, only 0.01%, 2%, and 0.0087% of all 1-, 2-, and 3-models passed the threshold we used (PCC > 0.1) as the best 1-, 2-, and 3-models, respectively (Supplemental Figure S3). Second, the best models failed to predict randomly shuffled TSS entropy values. We randomly shuffled the TSS entropy values, and the PCC was calculated between predicted entropy and those shuffled entropies. In most cases (>95%), the predictive PCCs were less than 0.1(Figure S3).

**Bibliography**

Carninci P Kasukawa T Katayama S Gough J Frith MC Maeda N Oyama R Ravasi T Lenhard B Wells C et al. 2005. The transcriptional landscape of the mammalian genome. Science (New York, NY 309(5740): 1559-1563.

Carninci P, Sandelin A, Lenhard B, Katayama S, Shimokawa K, Ponjavic J, Semple CA, Taylor MS, Engstrom PG, Frith MC et al. 2006. Genome-wide analysis of mammalian promoter architecture and evolution. Nature genetics 38(6): 626-635.

Hoskins RA, Landolin JM, Brown JB, Sandler JE, Takahashi H, Lassmann T, Yu C, Booth BW, Zhang D, Wan KH et al. 2011. Genome-wide analysis of promoter architecture in Drosophila melanogaster. Genome research 21(2): 182-192.

Kawaji H, Frith MC, Katayama S, Sandelin A, Kai C, Kawai J, Carninci P, Hayashizaki Y. 2006. Dynamic usage of transcription start sites within core promoters. Genome biology 7(12): R118.

Schoenberg DR, Maquat LE. 2009. Re-capping the message. Trends Biochem Sci 34(9): 435-442.

Xu H, Handoko L, Wei X, Ye C, Sheng J, Wei CL, Lin F, Sung WK. 2010. A signal-noise model for significance analysis of ChIP-seq with negative control. Bioinformatics 26(9): 1199-1204.

Figure S1. Comparison between TSS entropy’s and STD’s ability to distinguish two Gaussian populations with a uniform noise background. The colored squares represent the -log(P-value) of the Student’s t-tests for 100 estimated A) TSS entropies and B) STDs between two Gaussian populations with σ = 1 and σ =15. The x-axis represents the number of reads sampled for the test, and the y-axis represents the percentage of reads from a uniform noise background.

Figure S2. Comparison between TSS entropy’s and STD’s ability to distinguish two Gaussian populations with a Gaussian noise background. The colored squares represent the -log(P-value) of the Student’s t-tests for 100 estimated A) TSS entropies and B) STDs between two Gaussian populations with σ = 1 and σ =15. The x-axis represents the number of reads sampled for the test, and the y-axis represents the percentage of reads from a Gaussian noise background.

Figure S3. Performance distribution of best 2-models for K562 cells. A) The performance of all 2-models and B) the performance of the best 2-models on predicting shuffled entropies.

Figure S4. The receiver operating characteristic (ROC) curve for the performance of models trained in GM12878 and applied in NHEK cells A) for CpG-related promoters and B) for nonCpG-related promoters.

Figure S5. Features selected for the best models. Stacked bars represent the distributions of selected feature types among nucleosome position (NU), histone modification levels (HM), and DNA sequence information (SEQ). Squares and diamonds represent the mean PCC and the mean BIC of the best models in the corresponding model categories, respectively. (A, C, and E) CpG-related promoters; (B, D, and F) non-CpG-related promoters.

**Figure S6**. Predictive power of 2-models. The dots represent predictive power of models, blue and red indicate the models were trained and tested in CpG-related and nonCpG-related promoters, respectively. The models involved two histone modifications, one is from Class I, and the other is from Class II. x-axis is the PCC generated by the 2-models which one histone modifications is active and the other one is repressive in exons (H3K36me3 and H4K20me1); y-axis is the PCC generated by the 2-models which both histone modifications are active.

**Tables**

Table S1. The models of 1-, 2-, 3- and full-models.

Table S2. Two classes of histone modifications. The P-values for all histone modification types are less than 0.001.

Table S3. Clusters of Transcription factors.
